# Safety and immunogenicity of an mRNA-lipid nanoparticle vaccine candidate against SARS-CoV-2: A phase 1 randomized clinical trial

**DOI:** 10.1007/s00508-021-01922-y

**Published:** 2021-08-10

**Authors:** Peter G. Kremsner, Philipp Mann, Arne Kroidl, Isabel Leroux-Roels, Christoph Schindler, Julian J. Gabor, Mirjam Schunk, Geert Leroux-Roels, Jacobus J. Bosch, Rolf Fendel, Andrea Kreidenweiss, Thirumalaisamy P. Velavan, Mariola Fotin-Mleczek, Stefan O. Mueller, Gianluca Quintini, Oliver Schönborn‑Kellenberger, Dominik Vahrenhorst, Thomas Verstraeten, Margarida Alves de Mesquita, Lisa Walz, Olaf‑Oliver Wolz, Lidia Oostvogels, CV-NCOV-001 Study Group, Fien De Boever, Anniek Desimpel, Meral Esen, Ina Fischer, Judith Flügge, Otto Geisenberger, Christof Geldmacher, Katrin Held, Larissa Hoffmann, Michael Hölscher, Kristina Huber, Bart Jacobs, Jasper Joye, Jacqueline Kirschke, Norman Klopp, Erik Koehne, Carsten Köhler, Albert Lalremruata, Carlos Lamsfus-Calle, Le Thi Kieu Linh, Cathy Maes, Dafni Metaxa, Marie-Luise Molnar, Mariana Mueller, Gesine Müller-Schöner, Marion Quindel, Sabine Rappe, Liz Schultze-Naumburg, Carsten Schumacher, Sabine Schuster, Verena Thiel, Susanne Vejda, Gwenn Waerlop, Carola Westenberg, Katrin Wons, Andreas Zeder

**Affiliations:** 1 Institute of Tropical Medicine, University Hospital Tübingen, Tübingen, Germany; 2 German Centre for Infection Research (DZIF), partner site Tübingen, Tübingen, Germany; 3 Centre de Recherches Medicales de Lambarene, Lambarene, Gabon; 4 CureVac AG, Schumannstraße 27, 60325 Frankfurt, Germany; 5 Division of Infectious Diseases and Tropical Medicine, University Hospital, LMU Munich, Munich, Germany; 6 German Centre for Infection Research (DZIF), partner site Munich, Munich, Germany; 7 Ghent University Hospital, Ghent, Belgium; 8 Hannover Medical School (MHH), Hannover, Germany; 9 Vietnamese-German Center for Medical Research (VG-CARE), Hanoi, Viet Nam; 10 CureVac AG, Tübingen, Germany; 11 Cogitars, Heidelberg, Germany; 12 P95 Epidemiology and Pharmacovigilance, Leuven, Belgium

**Keywords:** S protein, Reactogenicity, COVID-19, Dose-response, Neutralizing antibodies

## Abstract

**Background:**

We used the RNActive® technology platform (CureVac N.V., Tübingen, Germany) to prepare CVnCoV, a COVID-19 vaccine containing sequence-optimized mRNA coding for a stabilized form of SARS-CoV‑2 spike (S) protein encapsulated in lipid nanoparticles (LNP).

**Methods:**

This is an interim analysis of a dosage escalation phase 1 study in healthy 18–60-year-old volunteers in Hannover, Munich and Tübingen, Germany, and Ghent, Belgium. After giving 2 intramuscular doses of CVnCoV or placebo 28 days apart we assessed solicited local and systemic adverse events (AE) for 7 days and unsolicited AEs for 28 days after each vaccination. Immunogenicity was measured as enzyme-linked immunosorbent assay (ELISA) IgG antibodies to SARS-CoV‑2 S‑protein and receptor binding domain (RBD), and SARS-CoV‑2 neutralizing titers (MN_50_).

**Results:**

In 245 volunteers who received 2 CVnCoV vaccinations (2 μg, *n* = 47, 4 μg, *n* = 48, 6 μg, *n* = 46, 8 μg, *n* = 44, 12 μg, *n* = 28) or placebo (*n* = 32) there were no vaccine-related serious AEs. Dosage-dependent increases in frequency and severity of solicited systemic AEs, and to a lesser extent local AEs, were mainly mild or moderate and transient in duration. Dosage-dependent increases in IgG antibodies to S‑protein and RBD and MN_50_ were evident in all groups 2 weeks after the second dose when 100% (23/23) seroconverted to S‑protein or RBD, and 83% (19/23) seroconverted for MN_50_ in the 12 μg group. Responses to 12 μg were comparable to those observed in convalescent sera from known COVID-19 patients.

**Conclusion:**

In this study 2 CVnCoV doses were safe, with acceptable reactogenicity and 12 μg dosages elicited levels of immune responses that overlapped those observed in convalescent sera.

**Supplementary Information:**

The online version of this article (10.1007/s00508-021-01922-y) contains supplementary material, which is available to authorized users.

## Introduction

Globally, there have been over 180 million confirmed cases of severe acute respiratory syndrome coronavirus 2 (SARS-CoV-2) infection and over 3.9 million deaths [1]. Many infections are asymptomatic or mild in severity, but respiratory distress in more severe cases requires mechanical ventilation in intensive care and can result in death [2]. Multiple research and development efforts have resulted in 104 human SARS-CoV‑2 vaccine candidates entering clinical testing with 184 in preclinical testing [3], with one approach being the use of mRNA coding for the appropriate target protein antigen [4].

CureVac has an established mRNA-based technology, RNActive® (CureVac N.V., Tübingen, Germany), for accelerated development of human vaccines [5]. Proof-of-concept was demonstrated in preclinical [6] and phase 1 studies [7, 8] using chemically unmodified mRNA coding for rabies virus glycoprotein (RABV-G) [6]. These led to development of formulations of mRNA encapsulated in lipid nanoparticles (LNP) and in the second phase 1 study two doses of 1 or 2 μg of RABV‑G mRNA-LNP elicited immune responses comparable to a three-dose regimen of a licensed rabies vaccine, and with acceptable tolerability [8].

The RNActive® technology platform has been applied to prepare CVnCoV, a SARS-CoV‑2 mRNA-LNP vaccine [9] targeting the glycosylated spike (S) protein on the viral surface, an essential component for SARS-CoV‑2 binding and uptake into mammalian cells [10, 11]. This process is dependent upon the receptor binding domains (RBDs) of the trimeric S protein [12, 13], and antibodies to the S protein RBD [14] or antibodies to S protein from convalescent COVID-19 patients [11] as well as protease inhibitors that inhibit S protein cleavage into its S1 and S2 subunits [12] were all protective in preclinical models. CVnCoV consists of LNP-encapsulated non-chemically modified mRNA with naturally occurring nucleotides encoding for a full-length S protein that includes two proline mutations (S-2P), previously shown to stabilize the conformation of the S proteins for MERS-CoV [15] and SARS-CoV [16], without affecting the furin cleavage site. The mRNA was codon-optimized to provide a high expression level of S protein and a moderate activation of innate immunity. In rodents CVnCoV induced neutralizing antibodies and T cell responses and provided broad lung protection in a hamster SARS-CoV-2-challenge model [15]. In rhesus macaques two 8 µg doses 4 weeks apart induced virus neutralizing titers that protected against replication of SARS-CoV‑2 and associated histopathological lesions in the upper respiratory tract following challenge [17].

We report a planned interim analysis of an ongoing, randomized, placebo-controlled phase 1 trial of a 2-dose schedule of CVnCoV 28 days apart to assess the safety, reactogenicity and immunogenicity of increasing dosages, from 2 µg to 12 µg, in healthy adults (18–60 years) who were either SARS-CoV-2-naïve or previously infected.

## Methods

For this ongoing, first in human, placebo-controlled, blinded, multicenter, phase 1 study we enrolled healthy adults in Hannover, Munich and Tübingen, Germany, and Ghent, Belgium. The study protocol was approved by the appropriate investigational review boards (IRB) and national regulatory authority for each site and was registered with ClinicalTrials.gov (Identifier: NCT 04449276). All study procedures were conducted according to ICH and GCP guidelines. Participants provided written informed consent at enrolment. The study was monitored by an internal safety review committee (iSRC) and a data safety monitoring board (DSMB) composed of independent external vaccine experts.

The primary objective was the evaluation of safety and reactogenicity of 1 or 2 intramuscular doses of different dosages of CVnCoV administered 28 days apart. Main secondary objectives were evaluations of humoral immune responses as SARS-CoV‑2 S protein-specific IgG and RBD IgG (ELISA) antibodies and SARS-CoV‑2 virus neutralizing antibodies. A subset of participants seropositive for SARS-CoV‑2 were also included in the study to assess whether the baseline serostatus impacted any of the assessed parameters.

### Participants

Eligible participants were adults of either sex who were in good health based on medical history and examination at screening, enrolled in two equal age groups (18–40 years and 41–60 years). Participants were screened for seropositivity to the SARS-CoV‑2 nucleocapsid antigen (N-antigen) for post hoc analysis of the impact of prior exposure. Main inclusion criteria were a body mass index (BMI) ≥ 18.0 kg/m^2^ and ≤ 30.0 kg/m^2^ and being available for the duration of the study. Main exclusion criteria were a known elevated risk of exposure to SARS-CoV‑2 infection (e.g. healthcare personnel directly involved in patient care or long-term care), or any history of COVID-19 infection or exposure to a COVID-19 infected individual within 2 weeks prior to the study, except for a subset of SARS-CoV‑2 seropositive subjects who were enrolled at each dosage level. Exclusion criteria applied to all participants included any current or history of immunosuppressive disorder or treatment, any previous confirmed infection with SARS or Middle East Respiratory Syndrome virus (MERS), or any known allergy to a vaccine component. Also excluded were those who were active smokers within the previous year, pregnant or breastfeeding women, and sponsor or study site employees or relatives. Women of child-bearing potential were required to have a negative pregnancy test within 3 days before receiving their first vaccination and to use an approved form of contraception from 1 month before the first vaccination until 3 months after the last vaccination.

### Study design

Dosage escalation started with 2 μg CVnCoV, progressively increasing in subsequent groups with 4, 6, 8, and 12 μg. Higher dosages of 16 and 20 μg are currently being investigated. For each dosage group there was a sentinel cohort of two COVID-19-naïve participants in each age group who were vaccinated open-label. After assessing 24 h of sentinel safety data the iSRC and DSMB chair approved vaccination of the next four participants in each age group (open-label, COVID-19-naïve participants) for that dosage group. After assessing 60 h safety data, the iSRC and DSMB approved vaccination of the remaining participants of that dosage group (including placebo participants and known SARS-CoV-2-seropositive participants, randomized and blinded) and sentinels of the next higher dosage group. This procedure was then repeated until the 8 µg group was vaccinated. For 12 µg, the procedure was the same, but open label without placebo control.

### Vaccine

CVnCoV is an investigational LNP-formulated RNActive® SARS-CoV‑2 vaccine composed of an mRNA that encodes a prefusion conformation-stabilized version of the full-length SARS-CoV‑2 spike (S) protein, and four lipid components: cholesterol, 1,2-distearoyl-sn-glycero-3-phosphocholine (DSPC), PEG-ylated lipid and a cationic lipid. Placebo was 0.9% NaCl. Each dose was administered by intramuscular injection in the deltoid.

### Safety assessments

Participants remained under direct supervision of site personnel for 4 h following vaccination, and then recorded in diary cards solicited local (injection site pain, redness, swelling, and itching) and systemic (headache, fatigue, chills, myalgia, arthralgia, nausea/vomiting, and diarrhea) adverse events and daily temperature (using a supplied thermometer) for 7 days after each vaccination. Unsolicited adverse events were recorded until 28 days after vaccination. All solicited AEs were graded for severity as grade 1 (mild), grade 2 (moderate) and grade 3 (severe) using the FDA grading scale [18] (see Supplementary material pages 2 and 3). Investigators reviewed the severity gradings and assessed causality as either related or unrelated. Laboratory safety assessments done on blood drawn on days 1, 2, 8, 30 and 36 were graded according to the FDA grading scale [18].

Serious adverse events (SAE) were to be reported to the investigator immediately, who notified the sponsor. Monitored adverse events of special interest (AESI) included potential immune-mediated diseases and COVID-19 disease. In the event of a confirmed COVID-19 infection the participant or treating healthcare provider were to complete a specific diary card. Safety monitoring is ongoing until 1 year after the last vaccination.

### Immunogenicity assessments—IgG ELISA

Sera obtained before each of the two vaccinations on days 1 and 29, and on days 8, 15, 36, 43 and 57 for immunogenicity assessments were stored at −80 °C before shipping on dry ice for measurement of the immune responses in accordance with EMA “Guideline on bioanalytical method validation” at Vismederi S.r.l., Siena, Italy. Anti-SARS-CoV-2-specific IgG levels were measured by enzyme-linked immunosorbent assay (ELISA) against S‑protein or RBD (see Supplement page 4). Titers were determined as the reciprocal of the highest serum dilution that is over the predetermined cut-off OD value (limit of detection plus matrix effect) and reported as geometric mean titers (GMT) of duplicates. If no antibody was detectable (all dilutions below cut-off OD), an arbitrary titer of 50 (half of the limit of quantification) was reported.

### Immunogenicity assessments—Neutralizing activity

SARS-CoV‑2 virus neutralization titers were determined by a microneutralization assay with cytopathic effect (CPE) read out [19] (see Supplement page 4). The neutralization titer (MN_50_) was the reciprocal of the highest serum dilution that protected more than the 50% of cells from CPE and reported as geometric mean titer (GMT) of duplicates. If no neutralization was observed, an arbitrary titer value of 5 (half the limit of quantification) was reported.

### Reference human convalescent sera

A pool of human COVID-19 convalescent sera consisting of 68 samples collected mainly 4–8 weeks after diagnostic confirmation of SARS-CoV‑2 infection was either purchased from MTG Group (Van Nuys, CA, USA) or donated by the Universitätsklinikum, Tübingen. Samples included 19 sera from hospitalized patients aged 25–74 years (mean age ± SD, 49.5 ± 15.1 years), and 49 sera from patients aged 18–66 years (mean age ± SD, 39.2 ± 13.1 years) who were not hospitalized but manifested clear COVID-19 illness with multiple symptoms.

### Statistics

This is an adaptive Bayesian dose escalation design with expansion arms, so the sample size was not based on any hypothesis but was intended to allow estimation of the probability that the true rate of adverse reactions for each dose lies in an acceptable safety range. A minimum of 12 evaluable participants per dose for the dose escalation followed by observer-blinded randomized expansion cohorts stratified by age group and baseline serology was considered adequate for this purpose but in anticipation of drop-outs and with uncertainty about the proportion which would subsequently be found to have prior asymptomatic exposure to SARS-CoV‑2 a conservative number of 48 per group (24 in each age group) was chosen.

Safety data were analyzed in the safety set composed of all those who received at least one study administration (vaccine or placebo) and had any postvaccination safety data available. Safety data are presented descriptively as numbers of participants and percentages of each group with a specific solicited AE, together with severity. SAEs are described by case. Primary endpoints for the safety objective were frequencies of SAEs, frequencies and severity of solicited AEs within 7 days of vaccination, and occurrence, intensities and causality of unsolicited AEs with 28 days of vaccination. In this interim report we distinguish between dosage groups and baseline serostatus, but not between age groups.

Immunogenicity was analyzed in all participants who received both vaccinations and who had no protocol deviation. Secondary immunogenicity endpoints include proportions of participants seroconverting with IgG (ELISA) antibodies against SARS-CoV‑2 S protein or RBD, and virus neutralizing titers. Data are presented as group median titers (with 25th and 75th percentiles) of individual antibody GMTs, and seroconversion rates (SCR) defined as group proportions demonstrating fourfold increases in titer over baseline. To analyze translation of IgG antibodies into neutralizing activity, the ratios between ELISA S‑protein and RBD IgGs to MN_50_ were generated for each participant and visit individually. Medians calculated per dose and visit are compared with human COVID-19 convalescent sera.

## Results

### Demographics

From 18 June 2020 to database lock on 5 November 2020 for this interim analysis, we enrolled and randomly allocated 245 adults to the different study groups (Table 1). Mean age overall was 40 years (SD 14), there were 141 (58%) men and 104 (42%) women, with a mean BMI of 25 kg/m^2^ (SD 2.6), the majority of whom were described as white (235, 96%). These demographics were consistent across the vaccine and placebo groups. The 40 (16%) participants seropositive for SARS-CoV-2 N-antigen were evenly distributed across groups.

**Table 1:** Demographics of the enrolled study population included in this interim analysis with known seropositivity for SARS-CoV-2 N antigen by group

	Group	1	2	3	4	5	6
		2 μg	4 μg	6 μg	8 μg	12 μg	Placebo
	*N* =	47	48	46	44	28	32
*Age *(years)	Mean	38.2	39.1	37.9	38.0	37.4	40.1
	SD	12.5	13.2	12.6	13.2	13.5	13.5
	Range	(18–60)	(19–59)	(20–59)	(20–59)	(19–59)	(19–60)
18–40 years, *n* =		24	24	24	24	17	16
41–60 years, *n* =		23	24	22	20	11	16
*Male*	*n (%)*	*27 (57)*	*25 (52)*	*31 (67)*	*26 (59)*	*17 (61)*	*15 (47)*
*Female*		*20 (43)*	*23 (48)*	*15 (33)*	*18 (41)*	*11 (39)*	*17 (53)*
*BMI* (kg/m^2^)	Mean	23.6	24.2	24.4	23.7	23.6	23.1
	SD	(2.54)	(2.76)	(2.71)	(2.64)	(2.56)	(2.48)
*Immune status n* (%)							
Seropositive		8 (17)	8 (17)	6 (13)	6 (14)	4 (14)	8 (25)
Seronegative		39 (83)	40 (83)	40 (87)	38 (86)	24 (86)	24 (75)

**Fig. 1:**
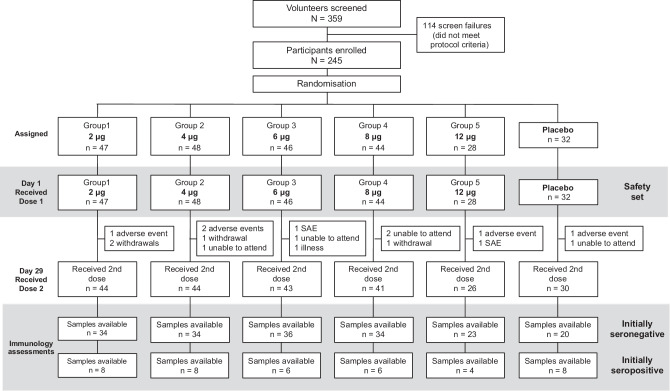
CONSORT study flow chart

As shown (Fig. 1) 228 (93%) participants received their second dose. Of the 17 participants (15 vaccine, 2 placebo) who did not receive their second dose five were unable to attend the visit, four withdrew consent and were lost to follow up, two had SAEs and one an illness that prevented them attending, four had adverse events considered unrelated to vaccination (fatigue, eye pain, dizziness, tooth abscess), and one initially seropositive participant had potential allergic reaction (a mild macular rash on the legs back and chest 4 h after the first vaccination).

### Safety

The primary objective to demonstrate the safety of CVnCoV was shown as no vaccine-related serious adverse events (SAE) or adverse events of special interest (AESI) were reported, and no AE led to withdrawal from the trial up to day 43. There were three SAEs reported, none of which were considered to be related to vaccination (Table 2): one case of a complicated fracture of the humerus in a bicycle accident and one of abdominal pain, both in seronegative participants, and one ligament rupture with peroneal palsy and pressure-induced monoplegia of the right foot in a seropositive participant.

**Table 2:** Unsolicited AEs, SAEs, medically attended AEs and AESIs after any vaccination

	Relationship	2 μg	4 μg	6 μg	8 μg	12 μg	Placebo
	*N* =	47	48	46	44	28	32
Unsolicited	*Any*	*22* *(47)*	*32* *(67)*	*29* *(63)*	*28* *(64)*	*21* *(75)*	*14* *(44)*
	Related	7 *(15)*	19 *(40)*	15 *(33)*	18 *(41)*	12 *(43)*	4 *(13)*
SAEs	*Any*	*3 (1.2)*^*a*^					
	Related	*0*					
Medically attended AEs	Any	2 *(4.3)*	1 *(2.1)*	4 *(8.7)*	3 (*6.8)*	4 *(14.3)*	6 *(18.8)*
AESI	Any	0	0	0	0	0	0

Italicised numbers are percentages

^a^The 3 unrelated SAEs are not shown by group to maintain blind for this interim analysis

**Fig. 2:**
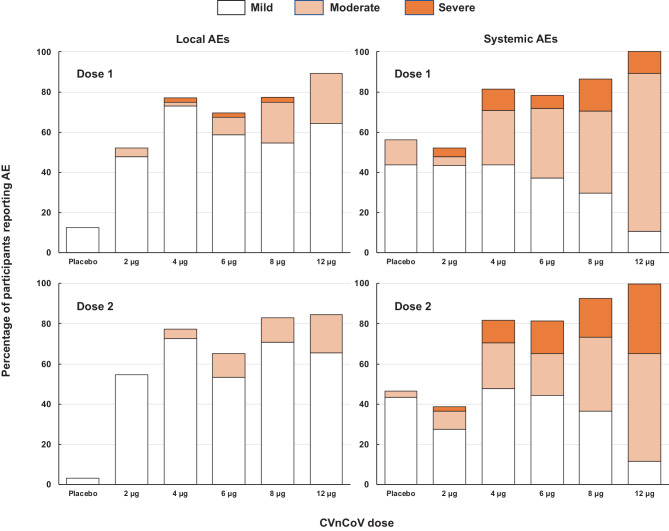
Overall incidence rates (%) of solicited local and systemic AEs per group with severity after the first and second doses

Overall, in SARS-CoV-2-naïve participants there was a dose-dependent increase in incidence and severity of local solicited AEs (Fig. 2). The vast majority of these reports were of grades 1 and 2 injection site pain (see Supplementary table 3); instances of severe pain usually had onset within 24 h of vaccination before decreasing in severity and resolving with 48 h. The incidence of reactions was similar after the second dose, but overall severity was lower as no grade 3 reactions were reported. Cases of swelling and itching were infrequent. The frequency and severity of solicited systemic adverse events increased with dosage, and 100% of the 12 μg group reported at least one solicited systemic AE (Fig. 2). Systemic AEs displayed similar overall rates after the first and second vaccinations, but severity increased after the second dose in the 4–12 μg dosage groups, e.g. 3 of 28 (11%) 12 μg recipients had grade 3 systemic AEs after the first dose compared with 9 of 26 (35%) after the second dose. Most grade 3 systemic AEs had decreased in severity or resolved within 24 h, and all did so with 72 h. The most frequent solicited systemic AEs were headache (39–88% of vaccine groups and 33% of placebo recipients), and fatigue (34–88% of vaccinees and 42% of placebo recipients) after the first doses (Supplementary table 4). Rates were similar after the second vaccinations, but proportionally more were described as severe (Supplementary table 4b). Fever was observed less frequently, in 2–38% and 3–52% of seronegative vaccinees after first and second doses, respectively, but with more grade 3 fevers after the second dose.

In participants who were SARS-CoV‑2 seropositive at the time of vaccination the local and systemic reactogenicity profiles were similar to the SARS-CoV-2-naïve participants. In SARS-CoV‑2 seropositive participants mild pain was reported by 50–100% across dose groups, with only two reports of moderate pain (8 and 12 μg), and the systemic reactogenicity profile was similar to the SARS-CoV-2-naïve participants, but with a lower frequency of grade 3 events after both doses (see Supplementary tables 4a and 4b). Of 32 initially seropositive vaccinees only one 4 µg group participant reported grade 3 solicited AEs (headache and fatigue).

Unsolicited AEs were reported by most participants in all groups (Table 2) and about half of which were considered study related. The most frequent unsolicited AE was dizziness, reported in 17 vaccinees across all groups and one placebo recipient. Many unsolicited AEs were the same types of event as solicited AEs, such as headache or fatigue, but which started after the 7‑day solicitation period.

Laboratory abnormalities were rare and showed no specific pattern, except for transient lymphopenia that was observed the day after vaccination in most participants of a small subset with available data from day 2. This has also been reported after influenza vaccination [20] and is thought to represent lymphocyte redistribution related to the mode of action of the vaccine (data not shown) [21].

### Immunogenicity

**Fig. 3:**
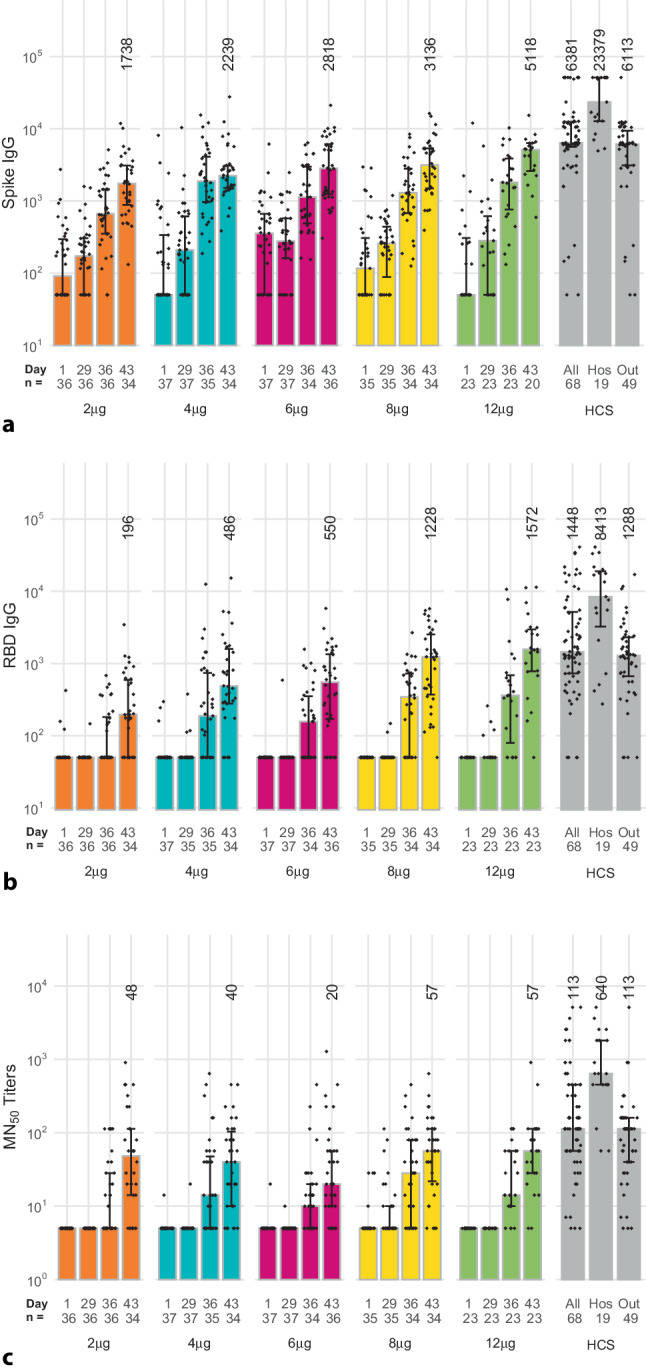
**a** Anti-spike protein IgG in initially seronegative participants who received both vaccinations in the different study groups, and human convalescent sera (HCS) samples (*Hos* hospitalized, *Out* outpatients) measured by ELISA. Bars show median values per group at each study timepoint (whiskers show IQR) and individual GMT values for each sample shown as diamonds. Numbers show median values at day 43 for each group, and in the convalescent sera. **b** Anti-RBD IgG in initially seronegative participants who received both vaccinations in the different study groups and human convalescent sera (HCS) samples (*Hos* hospitalized, *Out* outpatients) measured by ELISA. Bars show median values per group at each study timepoint (whiskers show IQR) and individual GMT values for each sample shown as diamonds. Numbers show median values at day 43 for each group, and in the convalescent sera. **c** Anti-SARS-CoV‑2 virus neutralizing titers in initially seronegative participants who received both vaccinations in the different study groups and human convalescent sera (HCS) samples (*Hos* hospitalized, *Out* outpatients) measured by microneutralization. Bars show median values per group at each study timepoint (whiskers show IQR) and individual GMT values for each sample shown as diamonds. Numbers show median MN_50_ values at day 43 for each group, and in the convalescent sera

Robust immune responses were observed in all groups of initially seronegative participants, with median titers comparable with those in sera from patients convalescing after COVID-19 infection (Fig. 3a–c). As no consistent changes were observed in median values in placebo recipients over the period studied, they are not included in the following descriptions. Several baseline samples had small but variable titers of ELISA IgG antibodies reactive to S protein (Fig. 3a). At day 29, 4 weeks after the first dose, there were small dose-dependent increases with SCRs of 6–26% across vaccine groups (Table 3), with more marked increases in all groups on day 36, 7 days after the second dose, with 50–74% seroconverting. The SCR continued to increase to 69–95% at day 43 when median titers were 1738 (IQR: 725–3094), 2239 (2175–3079), 2818 (12–6086), 3135 (56–5349), and 5118 (485–6319), in 2, 4, 6, 8 and 12 μg groups, respectively. Notably, the 12 μg group value at day 43 was comparable to the median titer of 6381 (5400–12432) in convalescent sera.

**Table 3:** Seroconversion rates (≥ fourfold increase) in baseline seronegative participants in each group at each time-point, *n/N* (%)

	Placebo	2 μg	4 μg	6 μg	8 μg	12 μg
*S protein IgG*						
Day 8	0/22 *(0)*	0/36 *(0)*	1/37 *(3)*	0/34 *(0)*	1/32 *(3)*	0/23 *(0)*
Day 29	0/22 *(0)*	2/36 *(6)*	6/37 *(16)*	6/37 *(16)*	9/35 *(26)*	4/23 *(17)*
Day 43	0/20 *(0)*	27/34 *(79)*	27/34 *(79)*	25/36 *(69)*	27/34 *(79)*	19/20 *(95)*
*RBD IgG*						
Day 8	0/22 *(0)*	0/35 *(0)*	0/37 *(0)*	0/34 *(0)*	1/32 *(3)*	0/23 *(0)*
Day 29	0/22 *(0)*	0/36 *(0)*	1/35 *(3)*	1/37 *(3)*	0/35 *(0)*	1/23 *(4)*
Day 43	0/20 *(0)*	13/34 *(38)*	27/34 *(79)*	25/36 *(69)*	28/34 *(82*)	21/23 *(91)*
*S protein or RBD IgG*						
Day 8	0/22 *(0)*	0/35 *(0)*	1/37 *(3)*	0/34 *(0)*	2/32 *(6)*	0/23 *(0)*
Day 29	0/22 *(0)*	2/36 *(6)*	7/36 *(19)*	1/37 *(19)*	9/35 *(26)*	5/23 *(22)*
Day 43	0/20 *(0)*	27/34 *(79)*	31/34 *(91)*	30/36 *(83)*	32/34 *(94)*	23/23 *(100)*
*Virus neutralizing titers*						
Day 8	0/22 *(0)*	0/38 *(0)*	0/37 *(0)*	0/34 *(0)*	0/32 *(0)*	0/23 *(0)*
Day 29	0/22 *(0)*	0/36 *(0)*	1/37 *(3)*	0/37 *(0)*	5/35 *(14)*	0/23 *(0)*
Day 43	2/20 *(10)*	24/34 *(71)*	23/34 *(68)*	20/36 *(56)*	27/34 *(79)*	19/23 *(83)*

Italicised numbers are percentages

The ELISA IgG antibody titers against RBD (Fig. 3b) generally reflect the same dose-dependent profile as IgG titers against S protein, with substantial increases in titers 7 days (day 36) after the second dose when seroconversion rates were 17–65% (Table 3). There was a further increase by day 43 when the seroconversion rates were 82% and 91% in the 8 and 12 μg groups with median titers of 1228 (1325–2542) and 1572 (535–2971), respectively, comparable to the median of 1448 (726–5391) observed in convalescent sera.

These observations of IgG antibody responses to S protein and RBD correlated with SARS-CoV‑2 neutralizing titers, as shown in Fig. 3c. This response was less obviously dose-dependent from the available samples, but across the groups 31–59% had seroconverted at day 36 (7 days after the second dose) from baseline, increasing to 56–83% at day 43 (Table 3). At day 43 median MN_50_ in the 8 µg and 12 µg groups (57 MN_50_, 7–113 and 57 MN_50_, 28–113) overlapped with the range observed in convalescent sera which had a median titer of 113 MN_50_, 57–453.

Interestingly 2 of 20 placebo recipients had developed low MN_50_ levels by day 43. Since this is not reflected in increased titers of binding antibodies, this is unlikely to be due to natural exposure to SARS-CoV‑2 but is potentially an artifact.

In initially SARS-CoV-2-seropositive participants the lowest doses of CVnCoV, 2 μg or 4 μg induced increases in antibody titers against S protein (not shown) and RBD binding antibodies and VNT within 1 week after the first vaccination (see Supplementary Fig. 1). Median RBD titers increased from 204 (IQR: 87, 366) at day 1 to 2494 (1399, 3204) at day 8 in the eight seropositive participants who received a 2 μg dose of CVnCoV; in the 4 μg group the respective increase was from 183 (50, 2296) to 3737 (999, 6814). There was no further increase after the second dose and median titers at day 43 were 3017 (IQR: 1576, 5828) and 5107 (2772, 9889) in the 2 μg or 4 μg groups, falling within the same range as the seronegative participants after two 12 μg doses. In seropositive subjects median MN_50_ titers were 108 (IQR: 40, 339) and 273 (113, 386) at day 1 in the 2 μg or 4 μg groups (*n* = 8 in each), increasing to 679 (IQR: 453, 905) and 1093 (640, 1920) at day 8, respectively. After small further increases at day 36 following the second dose to 1545 (IQR: 773, 1810) and 1810 (1543, 3840) titers then remained stable at least up to day 43.

When we assessed the ratios between neutralizing activity (MN_50_) and IgG antibodies against S protein (*n* = 20) or RBD (*n* = 23) for the 12 μg dosage at day 43 with those observed in the convalescent sera (*n* = 68) the corresponding medians were comparable. Respective median ratios in vaccinees and convalescent sera were 1.4 × 10^−2^ and 2.2 × 10^−2^ for MN_50_ vs. S protein IgG, and 3.6 × 10^−2^ and 8.4 × 10^−2^ for MN_50_ vs. RBD IgG.

## Discussion

In this interim report of the phase 1 clinical trial of CureVac’s mRNA COVID-19 vaccine candidate, CVnCoV, in 18–60-year-old adults the vaccine appeared safe and to have an acceptable reactogenicity profile at all doses from 2 µg to 12 µg, including participants known to be SARS-CoV‑2 seropositive at baseline. Compliance with the vaccination schedule was high, although investigators decided not to administer a second dose to four participants due to AEs, and one participant withdrew after an AE. There were no vaccine-related SAEs and despite increasing incidence and severity of solicited AEs with increasing dosages, reactogenicity did not limit participants’ willingness to receive both doses.

Local reactions were almost exclusively cases of transient mild to moderate injection site pain with a median duration of 1 day; only 3 first doses of the 415 total administered doses of CVnCoV resulted in severe local pain. The frequency and severity of solicited systemic AEs increased with dosage level and were generally of higher intensity after the second dose than the first, most notably for headache and to a lesser degree for fever and chills. The same reactogenicity profile has been reported for other mRNA SARS-CoV‑2 vaccines [22–25]. Systemic AEs mainly consisted of transient mild or moderate headache and fatigue, and to a lesser extent myalgia and chills, with fever being observed less frequently. Severe solicited AEs decreased or rapidly disappeared, mostly within 24–48 h of onset. The reactogenicity profile, with limited fever but symptoms like fatigue, headache and chills, is probably associated with the postulated mechanism of action and induction of an innate immune response mediated by interferon and other immune-stimulatory cytokines. Th1 cytokines are important for development of T cell responses, CD4 T cell help is required for good induction of memory B cells. Moreover, such a T-helper cell type 1 (Th1) biased immune response is desirable for the development of a SARS-CoV‑2 vaccine, due to the hypothetical concern for immune-mediated disease enhancement observed in preclinical studies for other coronaviruses. An IFN type 1 signaling has been also described in COVID-19 patients as a critical pathway to control disease [26, 27]. Investigations of cellular immunity are ongoing for this study and subsequent studies with the selected dosage.

All investigated dosages elicited an immune response against SARS-CoV‑2. Some participants who were seronegative in SARS-CoV-2 N-antigen testing did display some anti-S protein IgG antibodies, which may suggest that there is some cross-reactivity with S proteins from other coronaviruses. Most cross-reactivity between SARS-CoV and SARS-CoV‑2 has been reported to be at sites on the S2 subunit [28]. There was no prevaccination background IgG targeting the RBD in the S protein S1 subunit suggesting this assay may be more specific for SARS-CoV‑2. Induction of an adaptive humoral immune response was demonstrated by the increase in neutralizing antibodies; 56–77% of participants achieved MN_50_ seroconversion 2 weeks after 2 doses of 2–8 μg and 83% after 2 doses of 12 μg. Neutralizing activity was associated with marked S protein-specific and RBD-specific IgG antibody responses; notably 100% of 12 μg recipients seroconverted to either S protein or RBD by day 43 (Table 3). The S protein IgG and VNT responses were low but detectable after the first vaccination, but all markedly increased within 7 days of the second vaccination indicating efficient priming by the first dose.

Since an imbalance between neutralizing versus binding antibodies could hypothetically lead to immune-mediated disease enhancement, we calculated the ratios of neutralizing and IgG antibodies to S protein and RBD in 12 μg vaccinees at day 43 and convalescent sera. As the ratios in vaccinees were very close to those in convalescent sera after natural infection we hypothesized that the CVnCoV mechanism of action mimics the natural immune response to RNA viruses.

CVnCoV was well tolerated in SARS-CoV‑2 seropositive participants in whom immune memory appeared to have been induced by the natural infection. Low doses of CVnCoV (either 2 or 4 μg) were able to induce greater than 10-fold increases in antibody titers within 1 week, even in participants with low baseline antibody titers, while there was little or no response in seronegative participants 1 week after the first dose. This is consistent with observations with other mRNA-based vaccines [29, 30]. Furthermore, a second vaccination in that population did not lead to a further increase in antibody titer, suggesting that persons with prior SARS-CoV‑2 infection might not benefit from additional vaccinations and could be limited to a single dose application as also recently discussed by others [30, 31].

Our data in a hamster model showed that a single vaccination with a low dose of CVnCoV adequately primed the animals and that a viral challenge rapidly boosted the neutralizing antibody response, comparable to vaccination with a prime-boost regimen of two doses of vaccine [9]. Furthermore, 2 doses of 8 μg of CVnCoV induced robust humoral and cellular responses in non-human primates (NHPs) which prevented viral replication in the lungs and protected animals against SARS-CoV‑2 challenge [17]. Similarly, there was evidence of immune priming of NHPs with two low dosages (0.5 μg) which were not in themselves protective. Together, these preclinical and clinical data indicate a functional immune response mimicking the natural responses to infection, including induction of memory resulting in rapid responses to the second vaccination in initially seronegative vaccinees. We are currently performing more analyses on T‑cell and B‑cell memory responses that will provide further information on the unique mechanism of action of this mRNA vaccine candidate as well as administering higher doses (16 and 20 μg) to investigate the boundaries of the safety window. Further assessments of these groups are foreseen, including analysis of cellular immunity, and with safety and persistence follow-up until at least 1 year postvaccination. With the recent emergence of mutations of the original SARS-CoV‑2 virus, with changes on the S protein leading to variants of concern (VoC) [32] further studies will need to ensure induced antibodies are cross-reactive with the predominant circulating VoC.

This study has evident limitations inherent in a phase 1 trial, the most obvious being the small numbers in each group which do not allow us to make definitive conclusions about dosage. Similarly, the relatively few participants who were initially seropositive for SARS-CoV‑2 do not allow definitive conclusion about the impact of prior exposure. Nonetheless, the dosage-dependent increases in reactogenicity and immunogenicity are sufficient to allow selection of the 12 μg dosage as probably the best compromise between these two factors—high immunogenicity with acceptable tolerability. Further investigations of the immune response are required, most notably of the cellular immune response, which are currently ongoing.

However, in view of the urgency for COVID-19 vaccines, and the observation of an acceptable reactogenicity profile with a strong immune response in the range of convalescent sera, the 12 μg dosage has been selected for further investigation in an ongoing phase 2b/3 efficacy and safety study (ClinicalTrials.gov Identifier: NCT04652102) for which enrolment of 36,500 participants was initiated on 11 December 2020.

## Supplementary Information

In Supplementary materials we provide the definitions of the different severity grades of solicited local and systemic adverse events, and the incidence of those adverse events in the different study groups according to baseline serostatus for SARS-CoV-2 infection. We also illustrate the immune responses to 2 and 4 µg doses in initially seropositive individuals.
