# Supplementary material for: Safety and immunogenicity of an mRNA-lipid nanoparticle vaccine candidate against SARS-CoV-2: A phase 1 randomized clinical trial
Source: Wien Klin Wochenschr. 2021 Aug 10;133(17-18):931–41. doi: 10.1007/s00508-021-01922-y (PMC8354521; doi:10.1007/s00508-021-01922-y)

**Supplementary materials**

**Table 1:** Intensity Grading for Solicited Local Adverse Events page 2

**Table 2:** Intensity Grading for Solicited Systemic Adverse Events page 3

**Immunogenicity assessments** (ELISA and neutralization assay) page 4

**Table 3:** Solicited Local Adverse Events after each dose page 5

**Table 4a:** Solicited Systemic Adverse Events after the first dose page 6

**Table 4b:** Solicited Systemic Adverse Events after the second dose page 8

**Figure 1:** Boosting of antibody responses in seropositive participants page 10

| **Table 1:** Intensity Grading for Solicited Local Adverse Events | | |
| --- | --- | --- |
| **AE** | **Grade** | **Definition** |
| Pain at injection site | 0 | Absent |
|  | 1 | Does not interfere with activity |
|  | 2 | Interferes with activity and/or repeated use of non-narcotic pain reliever > 24 hours |
|  | 3 | Prevents daily activity and/or repeated use of narcotic pain reliever |
| Redness | 0 | ≤ 2·5 cm |
|  | 1 | 2·5 – 5 cm |
|  | 2 | 5·1 – 10 cm |
|  | 3 | >10 cm |
| Swelling | 0 | ≤ 2·5 cm |
|  | 1 | 2·5 – 5 cm and does not interfere with activity |
|  | 2 | 5·1 – 10 cm or interferes with activity |
|  | 3 | > 10 cm or prevents daily activity |
| Itching | 0 | Absent |
|  | 1 | Mild, no interference with normal activity |
|  | 2 | Moderate, some interference with normal activity |
|  | 3 | Significant, prevents normal activity |

| **Table 2:** Intensity Grading for Solicited Systemic Adverse Events | | |
| --- | --- | --- |
| **Adverse Event** | **Grade** | **Definition** |
| Fever | 0 | <38°C |
|  | 1 | ≥38 – 38·4°C |
|  | 2 | ≥38·5 – 38·9°C |
|  | 3 | ≥39°C |
| Headache | 0 | Absent |
|  | 1 | Mild, no interference with normal activity |
|  | 2 | Moderate, some interference with normal activity and/or repeated use of non-narcotic pain reliever >24 hours |
|  | 3 | Significant; any use of narcotic pain reliever and/or prevents daily activity |
| Fatigue | 0 | Absent |
|  | 1 | Mild, no interference with normal activity |
|  | 2 | Moderate, some interference with normal activity |
|  | 3 | Significant, prevents normal activity |
| Chills | 0 | Absent |
|  | 1 | Mild, no interference with normal activity |
|  | 2 | Moderate, some interference with normal activity |
|  | 3 | Significant, prevents normal activity |
| Myalgia | 0 | Absent |
|  | 1 | Mild, no interference with normal activity |
|  | 2 | Moderate, some interference with normal activity |
|  | 3 | Significant, prevents normal activity |
| Arthralgia | 0 | Absent |
|  | 1 | Mild, no interference with normal activity |
|  | 2 | Moderate, some interference with normal activity |
|  | 3 | Significant, prevents normal activity |
| Nausea/ Vomiting | 0 | Absent |
|  | 1 | Mild, no interference with activity and/or 1 – 2 episodes/ 24 hours |
|  | 2 | Moderate, some interference with activity and/or >2 episodes/ 24 hours |
|  | 3 | Significant, prevents daily activity, requires outpatient i.v. hydration |
| Diarrhoea | 0 | Absent |
|  | 1 | 2 – 3 loose stools or <400 g/24 hours |
|  | 2 | 4 – 5 stools or 400 – 800 g/24 hours |
|  | 3 | 6 or more watery stools or >800 g/24 hours or requires outpatient i.v. hydration |
| i.v.= Intravenous | | |

**Immunogenicity assessments**

**Anti-SARS-CoV-2-specific IgG levels were measured by ELISA**. Briefly, plates were coated with 1µg/ml of SARS-CoV-2 Spike (Spike S1+S2 ECD-His Recombinant Protein, Sino Biological, Chesterbrook, PA, USA) or Spike RBD (Spike RBD-His Recombinant Protein, Sino Biological) recombinant protein. Blocking was performed in 5% milk. Coated plates were incubated with heat-inactivated (56°C for 30 min) human serum in a 1:2-fold serial dilution (starting at 1:100). Antigen-specific IgG detection was performed with goat anti-human IgG-HRP conjugate and tetramethyl benzidine (TMB) substrate (Bethyl Laboratories, Montgomery, Texas, USA) at OD 450 nm.

**SARS-CoV-2 virus neutralization titers were determined using a microneutralization assay with Cytopathic Effect (CPE)-read out**. In brief, heat-inactivated (56°C for 30 min) human serum was serially diluted 1:2 (starting at 1:10) and incubated with 10^2.7^ TCID_50_/ml tissue culture infective dose of wild type SARS-CoV-2 virus strain 2019-nCov/Italy-INMI1 at 37⁰C 5% CO_2_ for 1 hour. Afterwards, semi-confluent Vero E6 cells (ATCC) were incubated with the virus-serum mixtures at 37°C 5% CO_2_ for 3 days. Cells were assessed for virus-induced CPE by light microscopy. The neutralization titer (MN_50_) was the reciprocal of the highest serum dilution that protected more than the 50% of cells from CPE and reported as geometric mean titer (GMT) of duplicates.

| **Table 3.** Solicited local AEs with severity after one and two doses according to baseline serostatus for SARS-CoV-2 N antigen, n (%) | | | | | | | | | | | | | |
| --- | --- | --- | --- | --- | --- | --- | --- | --- | --- | --- | --- | --- | --- |
|  |  | **SARS-CoV-2 seronegatives** | | | | | | **SARS-CoV-2 seropositives** | | | | | |
|  | **Severity** | **2 μg** | **4 μg** | **6 μg** | **8 μg** | **12 μg** | **Placebo** | **2 μg** | **4 μg** | **6 μg** | **8 μg** | **12 μg** | **Placebo** |
|  | N = | 38 | 40 | 40 | 38 | 24 | 24 | 8 | 8 | 6 | 6 | 4 | 8 |
| **After first dose** | | | | | | | | | | | | | |
| **Pain** | **Any** | **16 (42)** | **30 (75)** | **28 (70)** | **30 (79)** | **21 (88)** | **2 (8)** | **8 (100)** | **6 (75)** | **4 (67)** | **4 (67)** | **4 (100)** | **1 (13)** |
|  | Mild | 14 (37) | 28 (70) | 23 (58) | 21 (55) | 15 (63) | 2 (8) | 8 (100) | 6 (75) | 4 (67) | 3 (50) | 3 (75) | 1 (13) |
|  | Moderate | 2 (5) | 1 (3) | 4 (10) | 8 (21) | 6 (25) | 0 | 0 | 0 | 0 | 1 (17) | 1 (25) | 0 |
|  | Severe | 0 | 1 (3) | 1 (3) | 1 (3) | 0 | 0 | 0 | 0 | 0 | 0 | 0 | 0 |
| **Redness** | **Any** | **0** | **0** | **0** | **0** | **0** | **0** | **0** | **0** | **0** | **0** | **0** | **0** |
| **Swelling** | **Any** | **0** | **3 (8)** | **0** | **0** | **1 (4)** | **0** | **0** | **0** | **0** | **0** | **0** | **0** |
|  | Mild | 0 | 3 (8) | 0 | 0 | 1 (4) | 0 | 0 | 0 | 0 | 0 | 0 | 0 |
| **Itching** | **Any** | **0** | **0** | **3 (8)** | **1 (3)** | **1 (4)** | **1 (4)** | **0** | **0** | **0** | **0** | **0** | **0** |
|  | Mild | 0 | 0 | 3 (8) | 1 (3) | 1 (4) | 1 (4) | 0 | 0 | 0 | 0 | 0 | 0 |
| **After second dose** | | | | | | | | | | | | | |
|  | **N=** | **36** | **37** | **37** | **35** | **23** | **22** | **8** | **7** | **6** | **6** | **3** | **8** |
| **Pain** | **Any** | **19 (53)** | **29 (78)** | **26 (70)** | **28 (80)** | **19 (83)** | **1 (5)** | **5 (63)** | **5 (71)** | **2 (33)** | **6 (100)** | **3 (100)** | **0** |
|  | Mild | 19 (53) | 27 (73) | 21 (57) | 23 (66) | 15 (65) | 1 (5) | 5 (63) | 5 (71) | 2 (33) | 6 (100) | 2 (67) | 0 |
|  | Moderate | 0 | 2 (5) | 5 (14) | 5 (14) | 4 (17) | 0 | 0 | 0 | 0 | 0 | 1 (33) | 0 |
|  | Severe | 0 | 0 | 0 | 0 | 0 | 0 | 0 | 0 | 0 | 0 | 0 | 0 |
| **Redness** | **Any** | **0** | **0** | **0** | **0** | **0** | **0** | **0** | **0** | **0** | **0** | **0** | **0** |
| **Swelling** | **Any** | **0** | **1 (3)** | **0** | **0** | **0** | **0** | **0** | **0** | **0** | **0** | **0** | **0** |
|  | Mild | 0 | 1 (3) | 0 | 0 | 0 | 0 | 0 | 0 | 0 | 0 | 0 | 0 |
| **Itching** | **Any** | **0** | **1 (3)** | **1 (3)** | **2 (6)** | **1 (4)** | **0** | **0** | **0** | **0** | **0** | **0** | **0** |
|  | Mild | 0 | 1 (3) | 1 (3) | 2 (6) | 1 (4) | 0 | 0 | 0 | 0 | 0 | 0 | 0 |

| **Table 4a.** Solicited systemic AEs with severity after a first dose according to baseline serostatus for SARS-CoV-2 N antigen, n (%) | | | | | | | | | | | | | |
| --- | --- | --- | --- | --- | --- | --- | --- | --- | --- | --- | --- | --- | --- |
|  |  | **SARS-CoV-2 seronegatives** | | | | | | **SARS-CoV-2 seropositives** | | | | | |
|  | **Severity** | **2 μg** | **4 μg** | **6 μg** | **8 μg** | **12 μg** | **Placebo** | **2 μg** | **4 μg** | **6 μg** | **8 μg** | **12 μg** | **Placebo** |
|  | N = | 38 | 40 | 40 | 38 | 24 | 24 | 8 | 8 | 6 | 6 | 4 | 8 |
| **Fever** | **Any** | **2 (5)** | **6 (15)** | **5 (13)** | **11 (29)** | **9 (38)** | **0** | **0** | **2 (25)** | **1 (17)** | **3 (50)** | **3 (75)** | **0** |
|  | Mild | 1 (3) | 3 (8) | 4 (10) | 8 (21) | 5 (21) | 0 | 0 | 1 (13) | 0 | 3 (50) | 1 (25) | 0 |
|  | Moderate | 1 (3) | 1 (3) | 1 (3) | 2 (5) | 3 (13) | 0 | 0 | 1 (13) | 1 (17) | 0 | 2 (50) | 0 |
|  | Severe | 0 | 2 (5) | 0 | 1 (3) | 1 (4) | 0 | 0 | 0 | 0 | 0 | 0 | 0 |
| **Headache** | **Any** | **15 (39)** | **21 (53)** | **22 (55)** | **28 (74)** | **21 (88)** | **8 (33)** | **3 (38)** | **5 (63)** | **2 (33)** | **3 (50)** | **4 (100)** | **1 (13)** |
|  | Mild | 12 (32) | 13 (33) | 11 (28) | 12 (32) | 6 (25) | 6 (25) | 3 (38) | 5 (63) | 2 (33) | 2 (33) | 1 (25) | 1 (13) |
|  | Moderate | 1 (3) | 7 (18) | 10 (25) | 15 (40) | 15 (63) | 2 (8) | 0 | 0 | 0 | 1 (17) | 3 (75) | 0 |
|  | Severe | 2 (5) | 1 (3) | 1 (3) | 1 (3) | 0 | 0 | 0 | 0 | 0 | 0 | 0 | 0 |
| **Fatigue** | **Any** | **13 (34)** | **24 (60)** | **31 (78)** | **28 (74)** | **21 (88)** | **10 (42)** | **5 (63)** | **5 (63)** | **3 (50)** | **3 (50)** | **4 (100)** | **3 (38)** |
|  | Mild | 11 (29) | 14 (35) | 18 (45) | 14 (37) | 9 (38) | 8 (33) | 4 (50) | 3 (38) | 1 (17) | 2 (33) | 1 (25) | 3 (38) |
|  | Moderate | 0 | 5 (13) | 11 (28) | 11 (29) | 10 (42) | 2 (8) | 1 (13) | 2 (25) | 2 (33) | 1 (17) | 3 (75) | 0 |
|  | Severe | 2 (5) | 5 (13) | 2 (5) | 3 (8) | 2 (8) | 0 | 0 | 0 | 0 | 0 | 0 | 0 |
| **Chills** | **Any** | **3 (8)** | **8 (20)** | **9 (23)** | **10 (26)** | **13 (54)** | **0** | **0** | **3 (38)** | **1 (17)** | **3 (50)** | **2 (50)** | **0** |
|  | Mild | 1 (3) | 6 (15) | 5 (13) | 7 (18) | 7 (29) | 0 | 0 | 2 (25) | 0 | 3 (50) | 1 (25) | 0 |
|  | Moderate | 2 (5) | 0 | 4 (10) | 2 (5) | 6 (25) | 0 | 0 | 1 (13) | 1 (17) | 0 | 1 (25) | 0 |
|  | Severe | 0 | 2 (5) | 0 | 1 (3) | 0 | 0 | 0 | 0 | 0 | 0 | 0 | 0 |
| **Myalgia** | **Any** | **6 (16)** | **14 (35)** | **19 (48)** | **18 (45)** | **16 (67)** | **2 (8)** | **2 (25)** | **4 (50)** | **2 (33)** | **4 (67)** | **3 (75)** | **3 (38)** |
|  | Mild | 4 (11) | 8 (20) | 13 (33) | 9 (24) | 10 (42) | 2 (8) | 2 (25) | 3 (38) | 2 (33) | 3 (50) | 2 (50) | 3 (38) |
|  | Moderate | 2 (5) | 5 (13) | 6 (15) | 6 (16) | 6 (25) | 0 | 0 | 1 (13) | 0 | 1 (17) | 1 (25) | 0 |
|  | Severe | 0 | 1 (3) | 0 | 3 (8) | 0 | 0 | 0 | 0 | 0 | 0 | 0 | 0 |
| **Arthralgia** | **Any** | **5 (13)** | **8 (20)** | **9 (23)** | **11 (29)** | **11 (46)** | **0** | **0** | **2 (25)** | **0** | **0** | **0** | **0** |
|  | Mild | 5 (13) | 6 (15) | 6 (15) | 5 (13) | 9 (38) | 0 | 0 | 2 (25) | 0 | 0 | 0 | 0 |
|  | Moderate | 0 | 2 (5) | 3 (8) | 5 (13) | 2 (8) | 0 | 0 | 0 | 0 | 0 | 0 | 0 |
|  | Severe | 0 | 0 | 0 | 1 (3) | 0 | 0 | 0 | 0 | 0 | 0 | 0 | 0 |
| **Nausea/ Vomiting** | **Any** | **2 (5)** | **4 (10)** | **2 (5)** | **6 (16)** | **8 (33)** | **1 (4)** | **1 (13)** | **1 (13)** | **0** | **0** | **0** | **0** |
|  | Mild | 1 (3) | 4 (10) | 2 (5) | 6 (16) | 6 (25) | 1 (4) | 1 (13) | 1 (13) | 0 | 0 | 0 | 0 |
|  | Moderate | 1 (3) | 0 | 0 | 0 | 1 (4) | 0 | 0 | 0 | 0 | 0 | 0 | 0 |
|  | Severe | 0 | 0 | 0 | 0 | 1 (4) | 0 | 0 | 0 | 0 | 0 | 0 | 0 |
| **Diarrhoea** | **Any** | **2 (5)** | **5 (13)** | **8 (20)** | **3 (8)** | **3 (13)** | **0** | **1 (13)** | **1 (13)** | **0** | **0** | **1 (25)** | **0** |
|  | Mild | 2 (5) | 5 (13) | 7 (18) | 3 (8) | 3 13) | 0 | 1 (13) | 1 (13) | 0 | 0 | 1 (25) | 0 |
|  | Moderate | 0 | 0 | 1 (3) | 0 | 0 | 0 | 0 | 0 | 0 | 0 | 0 | 0 |
|  | Severe | 0 | 0 | 0 | 0 | 0 | 0 | 0 | 0 | 0 | 0 | 0 | 0 |

| **Table 4b.** Solicited systemic AEs with severity after a second dose according to baseline serostatus, n (%) | | | | | | | | | | | | | |
| --- | --- | --- | --- | --- | --- | --- | --- | --- | --- | --- | --- | --- | --- |
|  |  | **SARS-CoV-2 seronegatives** | | | | | | **SARS-CoV-2 seropositives** | | | | | |
|  | **Severity** | **2 μg** | **4 μg** | **6 μg** | **8 μg** | **12 μg** | **Placebo** | **2 μg** | **4 μg** | **6 μg** | **8 μg** | **12 μg** | **Placebo** |
|  | N = | 36 | 37 | 37 | 35 | 23 | 22 | 8 | 7 | 6 | 6 | 3 | 8 |
| **Fever** | **Any** | **1 (3)** | **3 (8)** | **10 (27)** | **12 (34)** | **12 (52)** | **0** | **0** | **0** | **1 (17)** | **0** | **2 (67)** | **0** |
|  | Mild | 1 (3) | 2 (5) | 6 (16) | 6 (17) | 5 (22) | 0 | 0 | 0 | 1 (17) | 0 | 0) | 0 |
|  | Moderate | 0 | 0 | 3 (8) | 3 (9) | 4 (17) | 0 | 0 | 0 | 0 | 0 | 2 (67) | 0 |
|  | Severe | 0 | 1 (3) | 1 (3) | 3 (9) | 3 (13) | 0 | 0 | 0 | 0 | 0 | 0 | 0 |
| **Headache** | **Any** | **8 (22)** | **22 (59)** | **24 (65)** | **31 (89)** | **19 (83)** | **6 (27)** | **3 (38)** | **4 (57)** | **4 (67)** | **5 (83)** | **3 (100)** | **1 (13)** |
|  | Mild | 6 (17) | 13 (35) | 12 (32) | 16 (46) | 4 (17) | 5 (23) | 2 (25) | 3(43) | 3 (50) | 5 (83) | 1 (33) | 1 (13) |
|  | Moderate | 2 (6) | 10 (11) | 8 (22) | 10 (29) | 12 (52) | 1 (5) | 1 (13) | 0 | 1 (17) | 0 | 2 (67) | 0 |
|  | Severe | 0 | 10 (11) | 4 (11) | 5 (14) | 3 (13) | 0 | 0 | 1 (14) | 0 | 0 | 0 | 0 |
| **Fatigue** | **Any** | **9 (25)** | **21 (57)** | **24 (65)** | **31 (89)** | **21 (88)** | **10 (42)** | **4 (50)** | **4 (63)** | **3 (50)** | **3 (50)** | **3 (100)** | **2 (25)** |
|  | Mild | 6 (17) | 13 (35) | 18 (45) | 16 (46) | 9 (38) | 8 (33) | 3 (38) | 3 (43) | 3 (50) | 3 (50) | 1 (33) | 2 (25) |
|  | Moderate | 3 (8) | 4 (11) | 11 (28) | 10 (29) | 10 (42) | 2 (8) | 1 (13) | 0 | 0 | 0 | 2 (67) | 0 |
|  | Severe | 0 | 4 (11) | 2 (5) | 5 (14) | 2 (8) | 0 | 0 | 1 (14) | 0 | 0 | 0 | 0 |
| **Chills** | **Any** | **3 (8)** | **7 (19)** | **13 (35)** | **13 (37)** | **19 (83)** | **0** | **1 (13)** | **0** | **2 (33)** | **2 (33)** | **2 (67)** | **0** |
|  | Mild | 2 (6) | 3 (8) | 7 (19) | 6 (17) | 5 (22) | 0 | 1 (13) | 0 | 2 (33) | 2 (33) | 0 | 0 |
|  | Moderate | 0 | 3 (8) | 5 (14) | 4 (11) | 10 (44) | 0 | 0 | 0 | 0 | 0 | 2 (67) | 0 |
|  | Severe | 1 (3) | 1 (3) | 1 (3) | 3 (9) | 4 (17) | 0 | 0 | 0 | 0 | 0 | 0 | 0 |
| **Myalgia** | **Any** | **5 (14)** | **14 (38)** | **20 (54)** | **16 (46)** | **16 (70)** | **1 (5)** | **2 (25)** | **1 (14)** | **3 (50)** | **4 (67)** | **3 (100)** | **0** |
|  | Mild | 4 (11) | 8 (22) | 12 (32) | 7 (20) | 8 (35) | 1 (5) | 2 (25) | 1 (14) | 3 (50) | 2 (33) | 3 (100) | 0 |
|  | Moderate | 1 (3) | 4 (11) | 7 (19) | 7 (20) | 7 (30) | 0 | 0 | 0 | 0 | 0 | 0 | 0 |
|  | Severe | 0 | 2 (5) | 1 (3) | 2 (6) | 1 (4) | 0 | 0 | 0 | 0 | 0 | 0 | 0 |
| **Arthralgia** | **Any** | **1 (3)** | **5 (14)** | **12 (32)** | **12 (34)** | **10 (43)** | **0** | **0** | **3 (43)** | **1 (17)** | **0** | **1 (33)** | **0** |
|  | Mild | 1 (3) | 3 (8) | 6 (16) | 9 (26) | 6 (26) | 0 | 0 | 3 (43) | 1 (17) | 0 | 1 (33) | 0 |
|  | Moderate | 0 | 1 (3) | 4 (11) | 2 (6) | 2 (9) | 0 | 0 | 0 | 0 | 0 | 0 | 0 |
|  | Severe | 0 | 1 (3) | 2 (5) | 1 (3) | 2 (9) | 0 | 0 | 0 | 0 | 0 | 0 | 0 |
| **Nausea/ Vomiting** | **Any** | **1 (3)** | **5 (14)** | **6 (16)** | **8 (23)** | **5 (22)** | **1 (5)** | **0** | **1 (14)** | **0** | **0** | **1 (33)** | **0** |
|  | Mild | 1 (3) | 3 (8) | 4 (11) | 8 (23) | 4 (17) | 1 (5) | 0 | 1 (14) | 0 | 0 | 0 | 0 |
|  | Moderate | 0 | 2 (5) | 1 (3) | 0 | 1 (4) | 0 | 0 | 0 | 0 | 0 | 1 (33) | 0 |
|  | Severe | 0 | 0 | 1 (3) | 0 | 0 | 0 | 0 | 0 | 0 | 0 | 0 | 0 |
| **Diarrhoea** | **Any** | **0** | **2 (5)** | **5 (14)** | **1 (3)** | **2 (9)** | **2 (9)** | **0** | **0** | **0** | **0** | **0** | **3 (38)** |
|  | Mild | 0 | 2 (5) | 3 (8) | 3 (3) | 2 (9) | 2 (9) | 0 | 0 | 0 | 0 | 0 | 3 (38) |
|  | Moderate | 0 | 0 | 2 (5) | 0 | 0 | 0 | 0 | 0 | 0 | 0 | 0 | 0 |
|  | Severe | 0 | 0 | 0 | 0 | 0 | 0 | 0 | 0 | 0 | 0 | 0 | 0 |

**Figure 1.** Boosting of baseline antibody levels in individual seropositive participants vaccinated with either 2 μg (upper panel) or 4 μg (lower panel) of CVnCoV on days 1 and 29. RBD binding antibodies and SARS-CoV-2 neutralising MN_50_ titers were analysed at multiple time points. Lines show individual participants in both dose groups.


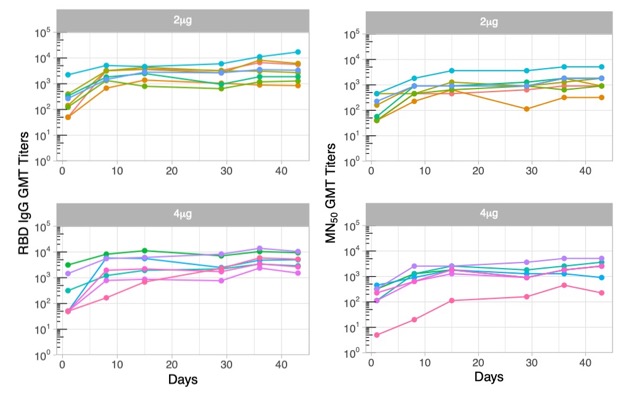

Supplement: Supplementary file 1 — In Supplementary materials we provide the definitions of the different severity grades of solicited local and systemic adverse events, and the incidence of those adverse events in the different study groups according to baseline serostatus for SARS-CoV-2 infection. We also illustrate the immune responses to 2 and 4 µg doses in initially seropositive individuals. [file 508_2021_1922_MOESM1_ESM.docx]
